# Molecular and epidemiological characterization of avian influenza viruses from gulls and dabbling ducks in Norway

**DOI:** 10.1186/1743-422X-10-112

**Published:** 2013-04-10

**Authors:** Ragnhild Tønnessen, Anja B Kristoffersen, Christine M Jonassen, Monika J Hjortaas, Elisabeth F Hansen, Espen Rimstad, Anna G Hauge

**Affiliations:** 1 Department of Food Safety & Infection Biology, Norwegian School of Veterinary Science, P. O. Box 8146 Dep N-0033, Oslo, Norway; 2 Norwegian Veterinary Institute, P. O. Box 750 Sentrum, N-0106, Oslo, Norway; 3 Present address: Norwegian Veterinary Institute, P. O. Box 750 Sentrum, N-0106, Oslo, Norway

**Keywords:** Avian influenza virus, Dabbling ducks, Gulls, Epidemiology, Reassortment

## Abstract

**Background:**

Wild aquatic birds constitute the natural reservoir for avian influenza viruses (AIVs). Separate Eurasian and American AIV gene pools exist. Here, the prevalence and diversity of AIVs in gulls and dabbling ducks in Norway were described. The influence of host species and temporal changes on AIV prevalence was examined. Five AIVs from Norway, including three from common gull (*Larus canus*), were analyzed along with 10 available AIV genomes from gulls in Eurasia to search for evidence of intracontinental and intercontinental reassortment of gene segments encoding the internal viral proteins.

**Methods:**

Swabs collected from 2417 dabbling ducks and gulls in the south-west of Norway during five ordinary hunting seasons (August-December) in the period 2005–2010 were analyzed for presence of AIV. Multivariate linear regression was used to identify associations between AIV prevalence, host species and sampling time. Five AIVs from mallard (*Anas platyrhynchos*) (H3N8, H9N2) and common gull (H6N8, H13N2, H16N3) were full-length characterized and phylogenetically analyzed together with GenBank reference sequences.

**Results:**

Low pathogenic AIVs were detected in 15.5% (CI: 14.1–17.0) of the samples. The overall AIV prevalence was lower in December compared to that found in August to November (p = 0.003). AIV was detected in 18.7% (CI: 16.8–20.6) of the dabbling ducks. A high AIV prevalence of 7.8% (CI; 5.9–10.0) was found in gulls. A similar temporal pattern in AIV prevalence was found in both bird groups. Thirteen hemagglutinin and eight neuraminidase subtypes were detected. No evidence of intercontinental reassortment was found. Eurasian avian (non H13 and H16) PB2 or PA genes were identified in five reference Eurasian gull (H13 and H16) AIV genomes from GenBank. The NA gene from the Norwegian H13N2 gull isolate was of Eurasian avian origin.

**Conclusions:**

The similar temporal pattern in AIV prevalence found in dabbling ducks and gulls, the relatively high virus prevalence detected in gulls and the evidence of intracontinental reassortment in AIVs from gulls indicate that gulls that interact with dabbling ducks are likely to be mixing vessels for AIVs from waterfowl and gulls. Our results support that intercontinental reassortment is rare in AIVs from gulls in Eurasia.

## Background

The natural reservoir for 16 hemagglutinin (HA) (H1-H16) and 9 neuraminidase (NA) (N1-N9) subtypes of influenza A viruses is wild aquatic birds, primarily ducks, gulls and shorebirds [1,2]. Influenza A viruses that cause epidemics and pandemics in humans, mammals and domestic poultry evolve directly or indirectly from the natural reservoir of avian influenza viruses (AIVs) [1].

Most AIVs are adapted to wild waterfowl and do not cause apparent clinical disease in their hosts. AIVs replicate in the intestinal and respiratory tract, but their tropism varies between bird species [3]. In ducks, virus transmission via the fecal-oral route through intake of virus-contaminated surface water is believed to be important [4], and infectious virus can persist in water and lake sediments for months [5,6]. In the northern hemisphere, the AIV prevalence in dabbling ducks is high in the fall, whereas a lower prevalence is found in the spring [7]. In gulls, the AIV prevalence is generally low [7-9], although higher virus prevalence has occasionally been detected in gull colonies [10,11].

Due to limited contact between birds from the different continents, AIVs have evolved into Eurasian and American phylogenetic lineages. However, this distinction is not absolute. In general, intercontinental reassortment of virus genes occurs relatively infrequently [12-20], but is more commonly detected in AIVs originating from areas of the world where migratory flyways overlap [14,15,18]. AIVs can also move with wild birds that cross the Atlantic Ocean [19,20]. Transmission of influenza virus genes between continents can lead to introduction and establishment of new virus lineages [19,21]. So far, intercontinental reassortment has involved exchange of single or multiple gene segments [19]. Transfer of complete AIV genomes has not been reported [14]. Within continents, exchange of virus genes is far more common due to frequent interspecies AIV transmissions that result in the formation of influenza viruses with transient genome constellations [16].

AIVs of the H13 and H16 subtypes are primarily restricted to gulls and shorebirds [2,22] and have originally been described to form separate American and Eurasian phylogenetic “gull-specific” lineages [8]. It was recently proposed to combine these two lineages into a “Charadriiformes specific” lineage due to the apparent high frequency of genetic reassortment occurring between viruses from these lineages [19,23]. Eurasian gene segments are commonly present in influenza A viruses isolated from gulls in North America, especially in the H13 and H16 subtypes, and it has therefore been suggested that long-distance migrating gull species play an important role in the transfer of AIV genes from Eurasia into America [18,19]. On the contrary, American gene segments have only been found on a few occasions in AIVs isolated from gulls in Eurasia [13,23]. However, the total number of fully sequenced AIV genomes from gulls in Eurasia is still low and more sequence information, particularly from coastal regions in Europe, has been requested [19].

Norway has the longest coastline in Europe and is located along the East-Atlantic flyway for migratory birds [8]. As a consequence of the emergence of highly pathogenic AIV subtype H5N1 in Southeast-Asia and its spread towards Europe, a surveillance program for avian influenza in wild birds was implemented in Norway in 2005 [24]. Except for a one-year gap in 2008, the program continued to 2010. In the present study, epidemiological analysis was performed on data obtained through five years of AIV surveillance. In addition, five virus isolates, including three from common gull (*Larus canus*) were sequenced. The aims of this study were to: i) describe the prevalence and subtype diversity of AIVs in dabbling ducks and gulls in the south-west (SW) of Norway, ii) test whether the virus prevalence was influenced by host species and sampling time, iii) determine the genetic similarity of the Norwegian AIVs to previously characterized AIVs, and to iv) search for evidence of intracontinental and intercontinental reassortment of gene segments encoding the internal viral proteins in AIVs from gulls in Eurasia.

## Methods

### Collection of samples

**Figure 1:**
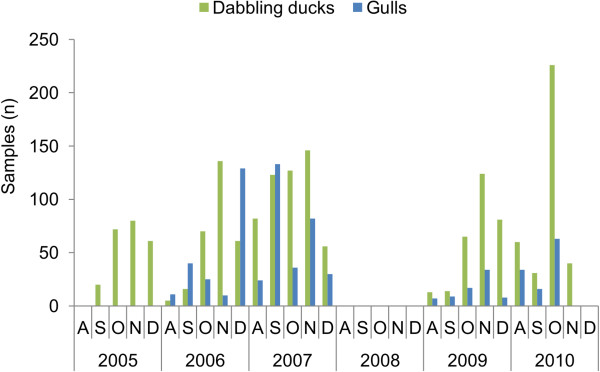
**Samples collected from wild birds in the south-west of Norway from 2005 to 2010.** Number of samples (n) collected each month from dabbling ducks and gulls in the south-west of Norway from 2005 to 2010. From 2005 to October 2006 cloacal swabs were collected, while from November 2006 and onwards, mixed cloacal and tracheal swabs were collected. No sampling was performed in 2008.

Swabs were collected by hunters from hunter-harvested dabbling ducks (n = 1709) and gulls (n = 708), in Rogaland County on the SW coast of Norway, during five licensed hunting seasons (2005–2007 and 2009–2010) from August to December. This is the most important area both for wintering wild aquatic birds and for poultry farming in Norway. The gulls were hunted regardless of the surveillance program. A part of the gulls were shot due to safety reasons to prevent bird strikes at the airport in Rogaland County, since the airport is situated in the middle of a wetland area. Others were shot since they were considered to be a problem for agriculture or in industrial and commercial areas. From each bird, cloacal and tracheal swabs were collected. Both swabs were placed in a single tube of virus transport medium (VTM) that was sent by postal mail to the Norwegian Veterinary Institute for analysis. The surveillance program for AIV was approved by the Norwegian Food Safety Authority (http://www.mattilsynet.no). An overview of the number of samples collected from August to December each year is presented in Figure 1.

### RNA extraction and rRT-PCR

Swabs collected from 2005 to 2007 were previously analyzed [24,25]. For samples collected in 2009 and 2010, RNA was isolated [25] and screened for AIV using real-time RT-PCR (rRT-PCR) targeting the matrix (M) gene, with modifications as described in [25]. If found AIV positive, an H5-specific rRT-PCR was performed [26]. Samples that tested positive for AIV, but negative for H5, were subtyped by sequencing of the HA2 part of the HA gene [27] and the full-length NA gene [28].

### Virus isolation and hemagglutination assay

Virus isolation was performed on a selection of AIV positive swabs. The samples, each containing one ml VTM, were thawed, vortexed, briefly centrifuged and 12.5–25 μl Gentamicin (50 mg/ml, Gibco, Paisley, UK) was added to the supernatant. After one hour incubation at room temperature, 100–200 μl medium was inoculated into the allantoic cavity of nine to 11 days old embryonated chicken eggs in three parallels and incubated for four days at 37°C. A minimum of two passages were made. The hemagglutination assay was performed using 1% chicken erythrocytes in a PBS de Boer solution [29]. The M gene specific rRT-PCR was used to confirm that the observed hemagglutination was caused by AIV [25]. Subtyping was performed as described above [27,28] and AIV positive allantoic fluid was stored at -80°C.

### Virus isolates

One H13, one H16 and one non-H13/H16 isolate from common gull were chosen for further characterization: A/common gull/Norway/1602/2009(H6N8), A/common gull/Norway/1313/2009(H13N2) and A/common gull/Norway/1617/2006(H16N3). In addition, two mallard (*Anas platyrhyncos*) isolates i.e. A/mallard/Norway/779/2009(H3N8) and A/mallard/Norway/1537/2009(H9N2) were randomly selected as references of Eurasian AIVs. Allantoic fluid from first (H6, H13), second (H3, H16) and third (H9) egg passages was used. The five virus isolates represent AIVs of subtypes frequently circulating in dabbling ducks and gulls in Norway.

### Sequencing of AIV genomes

For full-length amplification of the viral segments, a two-step RT-PCR was performed, using SuperScript® III (Invitrogen, Carlsbad, CA, USA) and the Advantage 2 PCR kit (Clontech, Mountain View, CA, USA). Primers [28] and cycling conditions [24] were used as previously described. As full-length amplification of all polymerase basic protein 1 (PB1) and polymerase basic protein 2 (PB2) genes was not successful, a new set of primers were designed for this purpose. The PCR amplicons were purified, using Zymoclean™ Gel DNA recovery kit (Zymo Research, Orange, CA), and cloned into pCR2.1 TOPO vectors according to the instructions of the manufacturer (Invitrogen). Sequencing was performed by using a commercial sequencing service (GATC Biotech, Constance, Germany). Primers for internal sequencing of the largest amplicons, PB2 and PB1, were designed. For each viral segment, three to six clones were sequenced. The sequences have been submitted to the EMBL Nucleotide Sequence Database (EMBL) [EMBL: HE802704-HE802743]. All primer sequences designed in this study are available upon request.

### Similarity searches and phylogenetic analysis

For the AIV genomes sequenced in this study, the sequences were assembled using Vector NTI Advance ver.11 (Invitrogen). Similarity searches were performed using the Basic Local Alignment Search Tool (BLAST) [30].

A search in the Influenza Virus Resource database at NCBI [31] for complete AIV genomes from gulls in Europe and Asia identified 13 genomes (as of February 2012) that could be included in the phylogenetic analysis along with the five virus genomes sequenced in the present study (Additional file 1: Table S1). Three virus isolates from the NCBI search were excluded from the analysis due to subtype (HPAI H5N1), suboptimal sequence quality (A/slaty-backed gull/Japan/6KS0191/2006(H4N8) or because it clustered in a separate genetic lineage very different from all the other sequences (A/slaty-backed gull/Japan/6KS0185/2006(H4N8)) [32].

To search for evidence of reassortment of the internal viral gene segments (PB2, PB1, PA, NP, M and NS), MEGA 4 was used to align the full-length nucleotide sequences by Clustal W and to create phylogenetic trees, using the Neighbour-Joining method with the Maximum composite likelihood model [33]. Bootstrap values of 1000 replicates were used to assess the nodal support. Reference sequences of American avian [GenBank: CY004904, CY004906-10, CY016144], American gull [GenBank: CY005070-71, CY005390, CY004562-63, CY003895, CY01498], Eurasian avian [GenBank: CY060363-65, CY060367, AB274986-87, CY060370] and Eurasian gull [GenBank: AY684874, AY684878, AY684883, M30753, EU580589, EU580578] AIVs were included in the analysis to evaluate the phylogeographical affiliation of the viral segments [20]. Eurasian and American gull AIVs were defined as AIVs of the H13 and H16 subtypes, whereas Eurasian and American AIVs were defined as all AIV subtypes, except H13 and H16 [8].

### Statistical analysis

Data (bird species and time of sampling) from AI surveillance in dabbling ducks and gulls in Rogaland from 2005 to 2007 [24,25] and from 2009 to 2010 were included in the statistical analysis. The overall AIV prevalence was estimated for each bird species and for the two bird groups; dabbling ducks and gulls. For these bird groups, the virus prevalence was also studied according to year and month. For each month, the data from 2005 to 2010 were pooled. Annual variations in AIV prevalence during fall (August to December) were studied in detail in three species of dabbling ducks i.e. mallard, common teal (*Anas crecca*) and Eurasian wigeon (*A. penelope*), and also in the two most sampled gull species, i.e. herring gull (*Larus argentatus*) and common gull. Descriptive statistics were performed using Excel 2007 (Microsoft, Redmond, WA). Furthermore, multivariate logistic regression was used to describe the associations between AIV prevalence and host species or bird group and year, month or sampling date and their intercepts. R [34] was used for this analysis and p-values larger than 0.05 were considered as significant. Models were compared using Akaike information criteria (AIC). Binomial 95% confidence intervals (CIs) were calculated for the AIV prevalence estimates.

## Results

### AIV prevalence and subtypes

**Figure 2:**
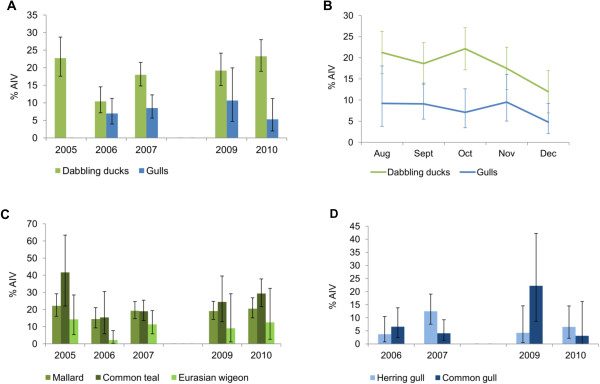
**Avian influenza virus (AIV) prevalence in dabbling ducks and gulls.** AIV prevalence from August to December according to **A**) year and **B**) month in 1709 dabbling ducks and 708 gulls sampled in the south-west of Norway. Samples were pooled according to month. The surveillance in gulls started in 2006. No samples were collected in 2008. Annual influenza A virus prevalence is also presented according host species, with mallard, common teal and Eurasian wigeon shown in **C**) and common gull and herring gull (n = 622) showed in **D**). Binomial 95% confidence intervals are indicated by the bars (**A-D**).

From 2005 to 2010 a total of 15.5% (CI: 14.1–17.0) of the 2417 birds tested positive for AIV by rRT-PCR (Table 1). Comparison of AIC values for different multivariate logistic regression models showed that bird group (dabbling ducks or gulls), year of sampling and December as sampling month or not were the variables that best described the AIV status of each bird. No interaction of the variables improved the AIC value, hence both bird groups showed similar pattern in AIV prevalence based on annual and monthly variation. Sampling date did not explain the data, probably due to clustered sampling periods. The prevalence of AIV was lower in December (p = 0.003) and varied among years with less number of AIV positive birds in 2006 (p < 0.001) and 2007 (p = 0.049) as compared to other years (Figures 2A,B). The overall AIV prevalence was higher in dabbling ducks than in gulls (p < 0.001) with 18.7% (CI: 16.8–20.6) and 7.8% (CI: 5.9–10.0), respectively.

**Table 1:** Prevalence of avian influenza virus (AIV) in dabbling ducks and gulls

**Species**	**Birds tested**	**AIV positive**	**AIV positive**	**CI**
	**n**	**n**	**%**	
DABBLING DUCKS				
Mallard (*Anas platyrhynchos*)	1008	194	19.2	16.9–21.8
Common teal (*Anas crecca*)	425	101	23.8	19.8–28.1
Eurasian wigeon (*Anas penelope*)	276	24	8.7	5.7–12.7
Total	1709	319	18.7	16.8–20.6
GULLS				
European herring gull (*Larus argentatus*)	349	28	8.0	5.4–11.4
Common gull (*Larus canus*)	273	18	6.6	4.0–10.2
Great black-backed gull (*Larus marinus*)	55	3	5.5	1.1–15.1
Black-headed gull (*Chroicocephalus ridibundus)*	25	6	24.0	9.4–45.1
Lesser black-backed gull (*Larus fuscus*)	5	0	0.0	0–52.2
Black-legged kittiwake (*Rissa tridactyla*)	1	0	0.0	0–97.5
Total	708	55	7.8	5.9–10.0
**Total**	**2417**	**374**	**15.5**	**14.1–17.0**

Pooled prevalence of AIV with 95% binomial confidence intervals (CIs) in dabbling ducks and gulls sampled during the hunting season (August to December) in the south-west of Norway from 2005 to 2010.

A multivariate logistic regression was also fitted to the dabbling ducks only. Duck species, December or not as sampling month and year gave the best model based on AIC value. Less birds were AIV positive in December (p = 0.008). Moreover, the AIV prevalence was lower in dabbling ducks in 2006 (p < 0.001) and 2007 (p = 0.027) than the other years. AIV was most frequently detected in common teal, closely followed by mallard, with no significant differences between the two species (Figure 2C, Table 1). The prevalence of AIV was significantly lower (p < 0.001) in Eurasian wigeon compared to that in both mallard and common teal (Table 1). The annual fluctuations in virus prevalence were similar for all the three species of dabbling ducks (Figure 2C). In gulls, the highest prevalence was detected in black-headed gull (*Chroicocephalus ridibundus*) (Table 1), however, the number of samples collected from this species was low. Most samples from gulls were collected from herring gull and common gull (Table 1). In herring gull and common gull, no significant difference in annual virus prevalence was observed (Figure 2D). A logistic regression with only these two gull species showed no significant difference between species, sampling date, month or year.

**Figure 3:**
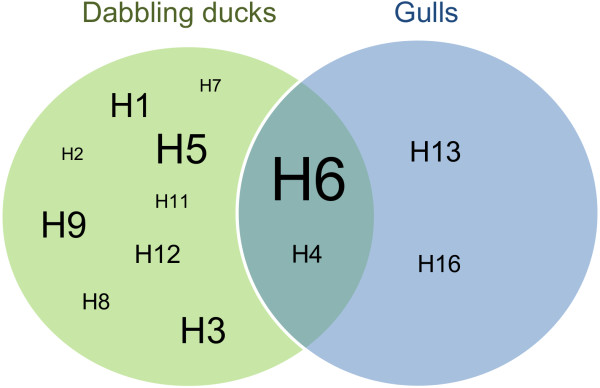
**Hemagglutinin (HA) subtypes of avian influenza virus detected in dabbling ducks and gulls.** HA subtypes of avian influenza virus detected in dabbling ducks and gulls during fall (August to December) in the south-west of Norway from 2005 to 2010. H6 was detected in 40.2% (86/214) (CI: 33.6–47.1) of the HA subtyped samples. The font size of the HA subtypes reflects their prevalence in the overall study population.

Of the 374 AIV positive samples, 223 were successfully subtyped. Of these, 105 samples were both HA and NA subtyped, whereas 109 and 9 samples were only HA or NA subtyped, respectively. Thirteen HA subtypes were detected. H6 was most frequently found, followed by H5, in 40.2% (CI: 33.6–47.1) and 13.1% (CI: 8.9–18.4) of the HA characterized samples, respectively. H3 and H9 were both found in 9.8% (CI: 6.2–14.6) of the samples. In dabbling ducks, H1-H9, H11 and H12 were detected, whereas in gulls, H4, H6, H13 and H16 were found (Figure 3). The H4 and H6 subtypes were found in both dabbling ducks and gulls (Figure 3). Phylogenetic analysis of the H4 and H6 genes showed no significant differences between the viruses from ducks and gulls (data not shown). The highest subtype diversity was found in mallard and common teal (data not shown). The H5 subtype was detected in 7.8% (CI: 5.3–10.9) (29/374) of the AIV positive samples and was found in mallard (n = 22) and common teal (n = 7). All H5 viruses had PQRETR/GLF or PQKETR/GLF HA cleavage sites and were therefore considered low pathogenic. H7 was only detected in one mallard, but its cleavage site was not determined. Eight NA subtypes (N1-N6, N8-N9) were detected. All of these were found in ducks, but only N1-N3, N6 and N8 were found in gulls. The N2 and N8 subtypes were most common and occurred in 66.7% (CI: 57.2–75.2) and 13.2% (CI: 7.6–20.1) of the NA subtyped samples, respectively.

### Similarity searches

The gene segments from the H3N8, H6N8 and H9N2 isolates were similar to Eurasian AIV gene segments (Additional file 2: Table S2). For the H13N2 and H16N3 viruses from common gull, the majority of genes were highly similar to Eurasian gull AIVs. The NA segment of the H13N2 virus was of Eurasian avian origin. For the PB2, NP and NA genes of the H16N3 virus, the results were more complicated to interpret, since highest identities were shared with the PB2, NP and NA genes of H13 and H16 AIVs isolated from American shorebirds containing gene segments previously proposed to have originated from Eurasian gull AIVs [18,19,35].

### Phylogenetic analysis

**Figure 4:**
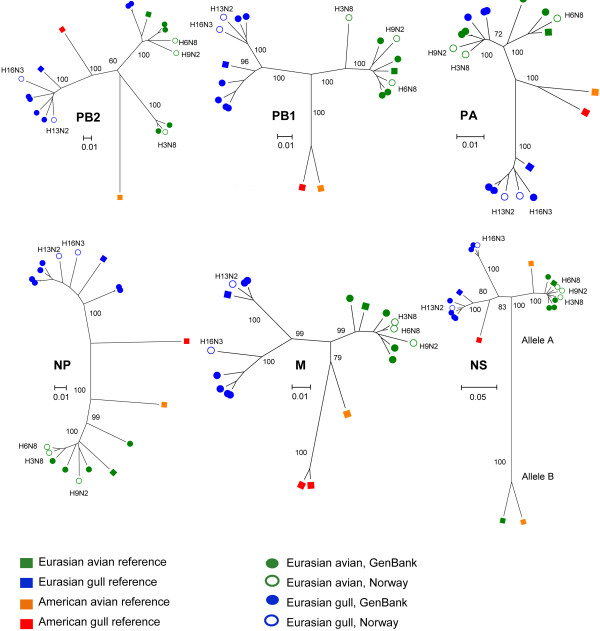
**Phylogenetic trees of the internal gene segments.** Phylogenetic trees of the PB2, PB1, PA, NP, M and NS avian influenza virus gene segments are shown. The phylogeny was inferred using the Neighbor-Joining method. The evolutionary distances were computed using the Maximum Composite Likelihood method. The scale bars show the number of base substitutions per site. The NS scale is different due to the large distance between allele A and B. The green, blue, yellow and red squares represent Eurasian avian, Eurasian gull, American avian and American gull reference sequences, respectively [19]. The closed green and blue circles indicate Eurasian avian and Eurasian gull sequences obtained from GenBank, whereas the open green and blue circles are Eurasian avian and Eurasian gull sequences from this study (Additional file 2: Table S2).

Phylogenetic analysis of the five virus isolates sequenced in this study showed that all internal gene segments were of Eurasian origin, and neither these isolates nor the reference AIVs from Eurasian gulls contained evidence of genetic reassortment between Eurasian and American AIVs (Figure 4). Evidence of reassortment between Eurasian avian and Eurasian gull AIVs was observed in two of the PB2 reference sequences [GenBank: GQ907309 and GQ907301], and in three of the PA reference sequences [GenBank: GQ907315, GQ907323 and JF775475]. No linkage between the reassortment of the PB2 and PA genes of these viruses was found. The Eurasian gull PB1, PA and M sequences were distinct from the American gull sequences and more closely related to the Eurasian avian sequences. In contrast, the evolutionary distance for American and Eurasian gull AIVs PB2, NP and NS was shorter, particularly for the NS gene (Figure 4). The NS genes of the five Norwegian virus isolates all belonged to allele A.

## Discussion

### AIV prevalence and subtypes

In this study, the AIV prevalence was concomitantly monitored in both dabbling ducks and gulls over several months and years in the SW of Norway. The overall AIV prevalence was 15.5%. Statistical analysis showed that the AIV prevalence was significantly lower in December than during the other months of sampling (August to November). It is not clear whether ducks are a source of AIV for gulls (or the opposite) or whether two separate transmission cycles occur in ducks and gulls, with only sporadic virus transmissions between these two groups of birds. Based on the multivariate analysis of our complete data set, no interactions i.e. between bird group and year or month, respectively, were found to be statistically significant. These results therefore suggest that the AIV prevalence follows the same annual and monthly patterns from August to December in both dabbling ducks and gulls. This further indicates that a certain exchange of AIVs between these two bird groups probably occurs during this period. The high virus prevalence observed during August to November each year is most likely a result of extensive interspecies mixing and virus transmission, combined with gathering of a large number of immunological naïve juvenile birds [7]. The relatively cold climate in Norway may also increase the environmental persistence of these viruses [5]. It is unknown if the individuals tested were resident, overwintering or migrating birds, and information about the age of the birds included in this study was not available.

Similar virus prevalences were found in common teal (23.8%) and mallard (19.2%), whereas a significantly lower AIV prevalence was found in Eurasian wigeon (8.7%). The largest subtype diversity was found in mallard and common teal, which is in accordance with previous reports [7]. In dabbling ducks in general, there were no significant differences in virus prevalence within the period August to November. Other studies have reported on a peak in AIV prevalence during October-November [7,25,36]. In dabbling ducks, host traits like surface-feeding and a high density of filtering plates on the internal surface of the beak increase the exposure to AIV [37]. Mallards, common teals and Eurasian wigeons are known to gather in large flocks during fall and this could facilitate interspecies transmission of virus. The Eurasian wigeon is more terrestrial compared to the mallard and the common teal, and this behavior could lower the risk of getting infected via virus-contaminated water for this species.

In this study, a relatively large number of gulls (n = 708) were tested for AIV, and the overall prevalence of AIV in gulls was high (7.8%) compared to prevalence values reported in other studies [7-9]. The AIV prevalence in gulls has previously been reported to peak in late summer and early fall [8]. One possible explanation for the high prevalence of AIV in gulls may be virus spill-over from ducks to gulls due to partially overlapping habitat use during fall. As mentioned above, our complete data set indicated that the AIV prevalence in dabbling ducks and gulls follows the same annual and monthly patterns from August to December. There was a tendency for a decrease in virus prevalence in December in gulls, however no statistically significant differences in AIV prevalence between months of sampling were found when analyzing the data set from gulls alone. The high virus prevalence in gulls could be caused by spill-over of H4 and H6 AIVs from ducks, as these subtypes were detected in both dabbling ducks and gulls. No significant genetic differences between the H4 and H6 viruses from ducks and gulls were found. The H6 subtype is known to have a broad host-range [7] and was the most frequently detected HA subtype in this study.

Thirteen HA subtypes, i.e. all except H10, H14, H15, and eight NA subtypes, i.e. all except N7, were identified. During the same period, H10 [25] and N7 were detected in other parts of Norway, whereas H14 and H15 have never been found (personal observations). In the present study, H5, H6, H9 and H3 were the most frequently detected HA subtypes, whereas N2 and N8 were the most commonly detected NA subtypes. Several attempts were made to subtype the samples from 2005 to 2007. In 2009 and 2010, only one attempt per sample was performed. In this study, a H5 rRT-PCR specific for detection of Eurasian H5 viruses was used [26] which means that it is uncertain whether this test could detect American H5 AIVs potentially introduced by migratory birds into Eurasia. However, the HA gene from positive samples that tested negative in the H5 specific rRT-PCR were amplified and sequenced using generic primers targeting HA2 [27] allowing for possible identification of H5 from American lineages. In addition, non-specific NA primers that amplify the N1, N2, N4, N5 and N8 genes were often the first choice used for NA subtyping instead of NA subtype specific primers that detect N3, N6, N7 and N9 [28]. This may have introduced a bias in the relative detection rate of the different HA and NA subtypes. However, the occurrence of the different HA and NA subtypes is in accordance with previous results from Northern Europe [7,13].

### Intercontinental reassortment

The similarity searches showed that the majority of gene segments from the five complete AIV genomes in our study were of Eurasian origin and that they were most similar to North European AIV sequences. The PB2, NP and NA genes of the Norwegian H16N3 isolate were most similar to AIV genes from American H13 and H16 viruses isolated from shorebirds and gulls. However, these American virus isolates have previously been described as reassortant viruses containing Eurasian gene segments [19,20,35]. The NA and NP genes from the Norwegian H16N3 isolate were also highly similar to the N3 and NP genes of A/glaucous gull/Alaska/44198-027/2006(H16N3), for which Ramey et al. (2010) were not able to determine certain continental affiliations [18]. The NA gene of the Norwegian H16N3 virus was also similar to A/glaucous-winged gull/Southcentral Alaska/9JR0783R0/2009(H16N3). Wille et al. (2011) reported that the N3 genes of the Alaskan gull viruses mentioned above were more similar to sequences from Eurasian viruses than to those from other gull viruses in America. However, they were still described as distinct from the most closely related Eurasian lineage, and this was suggested to be caused by few available H16N3 virus sequences or by the presence of a unique lineage of the N3 gene in western North America [19].

The phylogenetic analysis showed that for the PB1, PA and M genes, the Eurasian gull sequences were distinct from the American gull sequences and more closely related to the Eurasian avian sequences. In contrast, for PB2, NP and NS, the evolutionary distance between American and Eurasian gull AIVs was shorter, particularly for the NS gene. This confirms the findings reported by Fouchier et al. (2005) who found that influenza A viruses isolated from gulls could be distinguished from other AIVs based on the PB2, NP, and NS genes but not necessarily based on the PB1, PA, and M genes [2].

No evidence of intercontinental gene reassortment between Eurasian and American AIVs was found in the internal genes of the Eurasian gull viruses analyzed in the present study. These results confirm the findings by Wille et al. (2011), who found evidence of transcontinental gene transfer in American gull AIVs, but not in European gull AIVs [19]. The apparent one-way transfer of viral segments from Eurasia to America should be further studied in Eurasian gull AIV isolates, particularly from pelagic, far migrating gull species, since they are considered to be most important for intercontinental movement of AIV genes [19]. Recently, H6N1 and H5N2 AIVs that contained American avian M genes were isolated from herring gulls in Belgium [23]. The three virus isolates from gulls sequenced in this study were all from common gull. This species is not known to migrate across the Atlantic Ocean [38], and therefore, it might be a less likely long-distance AIV vector, compared to pelagic gull species like for instance the black-legged kittiwake (*Rissa tridactyla*) and the great black-backed gull (*Larus marinus*) [20,39]. Some long-distance migratory shorebird species within Scolopacidae, for example *Calidris* sp. and ruddy turnstone (*Arenaria interpres*), may also contribute to the intercontinental gene reassortment between American and Eurasian AIVs.

### Intracontinental reassortment

The similarity search showed that the N2 gene of the Norwegian H13N2 gull virus was most similar to Eurasian avian viruses, which indicates that intracontinental reassortment i.e. reassortment between a Eurasian avian and a Eurasian gull H13 AIV has occurred. A similar finding was reported in a H13N2 AIV, isolated from great black-backed gull in Newfoundland, where the NA gene was most similar to American avian viruses [20].

Eurasian avian PA and PB2 genes were observed in half of the Eurasian gull genomes obtained from GenBank. All reassortant viruses were previously described as Mongolian H13 and H16 viruses [40]. Recently, Eurasian avian PA segments were also observed in two H13N6 viruses isolated in Belgium from yellow-legged gull (*Larus michaellis*) and herring gull, respectively [23]. Together this indicates that multiple infections of gulls with both Eurasian avian and Eurasian gull viruses are common. Gulls that frequently interact with waterfowl probably act as mixing vessels for Eurasian avian and Eurasian gull AIVs. Alternatively, but maybe less likely, viruses mix in other species and spill-over back to gulls. Especially segment 3, encoding the polymerase protein PA, seems to be frequently transferred from Eurasian avian to Eurasian gull AIVs [23,40], which could indicate that H13 and H16 AIVs are not dependent of gull adapted PA genes to replicate.

## Conclusions

To summarize, a high AIV prevalence was found in both dabbling ducks and gulls in the SW of Norway. Multiple AIV subtypes were present within a relatively small geographical area. Only low pathogenic viruses were found and most viral segments were highly similar to the genes of contemporary Eurasian viruses. Intracontinental reassortment events were identified in H13 and H16 AIVs from gulls. A similar temporal pattern in AIV prevalence was found in dabbling ducks and gulls. This together with the high virus prevalence in gulls and the intracontinental reassortment suggests that gull species frequently interacting with dabbling ducks are likely to be mixing vessels for AIVs from waterfowl and gulls. Evidence of intercontinental reassortment was not found, which supports that these events are rare in Eurasian gull AIVs. However, this apparent one-way transfer of virus segments from Eurasia to America should be further studied.

## Competing interests

The authors declare that they have no competing interests.

## Authors’ contributions

RT, AGH, CMJ and ER were responsible for conception and design of the study. MJH, AGH and RT performed the initial PCR analysis and subtyping of the samples. RT and AGH were responsible for virus isolation and hemagglutination assays. RT, EFH and AGH cloned and sequenced the virus isolates. RT performed the similarity searches and the phylogenetic analysis. ABK and RT performed the statistical analysis. The article was drafted by RT, AGH, ER, CMJ, MJH, EFH and ABK. All authors read, revised and approved the final manuscript.

## Supplementary Material

Additional file 1: Table S1 AIV genomes analyzed in this study. Click here for file

Additional file 2: Table S2 Results from the BLAST similarity searches. Results from the BLAST similarity searches performed on the full-length nucleotide sequences from the five virus isolates (H3N8, H6N8, H9N2, H13N2 and H16N3) sequenced in this study. Sequence with maximum identities is shown in brackets. The squares are colored green for Eurasian avian-like and blue for Eurasian gull-like segments. Click here for file
