# Supplementary material for: Molecular and epidemiological characterization of avian influenza viruses from gulls and dabbling ducks in Norway
Source: Virol J. 2013 Apr 10;10:112. doi: 10.1186/1743-422X-10-112 (PMC3639200; doi:10.1186/1743-422X-10-112)
Supplement: Additional file 1: Table S1 — AIV genomes analyzed in this study. [file 1743-422X-10-112-S1.docx]

| **Virus isolates** | **GenBank accession numbers**  Segment 1-8 |
| --- | --- |
| A/mallard/Norway/779/2009(H3N8)^1^ | HE802728-35 |
| A/black-headed gull/Netherlands/1/2006(H4N5) | CY076992-99 |
| A/gull/Moscow/3100/2006(H6N2) | EU152234-41 |
| A/common gull/Norway/1602/2009(H6N8)^1^ | HE802704-11 |
| A/black-headed gull/Netherlands/1/2005(H6N8) | CY041378-85 |
| A/mallard/Norway/1537/2009(H9N2)^1^ | HE802720-27 |
| A/herring gull/Netherlands/4/2006(H10N4) | CY077032-39 |
| A/common gull/Norway/1313/2009(H13N2)^1^ | HE802712-19 |
| A/Mongolian gull/Mongolia/401/2007(H13N6) | GQ907310-17 |
| A/Mongolian gull/Mongolia/405/2007(H13N6) | GQ907318-25 |
| A/black headed gull/Mongolia/1766/2006(H13N6) | GQ907302-9 |
| A/black-headed gull/Sweden/1/2005(H13N8) | CY077000-7 |
| A/herring gull/Mongolia/454/2008(H13N8) | JF775470-77 |
| A/black headed gull/Mongolia/1756/2006(H16N3) | GQ907294-301 |
| A/common gull/Norway/1617/2006(H16N3)^1^ | HE802736-43 |

**Additional file 1 - Table S1. AIV genomes analysed in this study**

^1^Virus isolates sequenced in this study.
