# Supplementary material for: Molecular and epidemiological characterization of avian influenza viruses from gulls and dabbling ducks in Norway
Source: Virol J. 2013 Apr 10;10:112. doi: 10.1186/1743-422X-10-112 (PMC3639200; doi:10.1186/1743-422X-10-112)
Supplement: Additional file 2: Table S2 — Results from the BLAST similarity searches. Results from the BLAST similarity searches performed on the full-length nucleotide sequences from the five virus isolates (H3N8, H6N8, H9N2, H13N2 and H16N3) sequenced in this study. Sequence with maximum identities is shown in brackets. The squares are colored green for Eurasian avian-like and blue for Eurasian gull-like segments. [file 1743-422X-10-112-S2.pdf]

Additional file 2 - Table S2. Results from the BLAST similarity searches

Results from the BLAST similarity searches of the full length nucleotide sequences from the five virus isolates (H3N8, H6N8, H9N2, H13N2 and H16N3) sequenced in this study. Sequence with maximum identities is shown in brackets and is coloured green for Eurasian avian-like and blue for Eurasian gull-like. Gull-like = H13 and H16, Avian-like = all subtypes except H13 and H16.

	PB2	PB1	PA	HA
**H3N8**	**EU152234 (99)** A/gull/Moscow/3100/2006 (H6N2)	**CY060292 (99)** A/mallard/Sweden/48/2002 (H11N9)	**CY049771 (98)** A/garganey/Altai/1213/2007 (H5N2)	**JF682614 (99)** A/mallard/Czech Republic/14516/2007 (H3N8)
**H6N8**	**JF789607 (99)** A/mallard/Czech Republic/13577-24K/2010 (H3N8)	**DQ251450 (98)** A/duck/Denmark/65047/04 (H5N2)	**CY060438 (98)** A/whitefronted goose/Netherlands 2/1999 (H6N2)	**CY041402 (99)** A/mallard/Netherlands/11/2007 (H6N5)
**H9N2**	**HQ244427 (97)** A/mallard/Czech Republic/15902-17K/2009 (H6N2)	**GQ240811 (99)** A/mute swan/Hungary/5973/2007 (H7N7)	**JF682620 (99)** A/mallard/Czech Republic/15902-18K/2009 (H11N9)	**GU194481 (99)** A/mallard/Switzerland/WV1070805/2007 (H9N2)
**H13N2**	**JF775477 (96)** A/herring gull/Mongolia/454/2008 (H13N8)	**GQ907300 (97)** A/black headed gull/Mongolia/1756/2006 (H16N3)	**GQ907299 (98)** A/black headed gull/Mongolia/1756/2006 (H16N3)	**GQ907318 (99)** A/Mongolian gull/Mongolia/405/2007 (H13N6)
**H16N3**	**EU030974 (97)** A/shorebird/Delaware/168/06 (H16N3)	**GQ907300 (97)** A/black headed gull/Mongolia/1756/2006 (H16N3)	**AY684883 (98)** A/black-headed gull/Sweden/1/99 (H13N6)	**FM179756 (96)** A/herring gull/Norway/10_1623/2006 (H16N3)

Table S2 continues

	NP	NA	M	NS
**H3N8**	**CY043835 (99)** A/mallard/Netherlands/29/2006 (H7N2)	**JF682616 (99)** A/mallard/Czech Republic/14516/2007 (H3N8)	**GQ404573 (99)** A/mallard Czech Republic/15307-17/2008	**CY076972 (99)** A/mallard/Netherlands/20/2005 (H12N8)
**H6N8**	**CY043835 (99)** (A/mallard/Netherlands/29/2006 (H7N2)	**FN773071 (99)** A/Teal/Norway/10_1575/2007 (H3N8)	**GQ404573 (99)** A/mallard Czech Republic/15307-17/2008	**HQ244411 (99)** A/Anas crecca/Spain/1460/2008 (H7N9)
**H9N2**	**CY029884 (97)** A/sharp-tailed sandpiper/Australia/10/2004 (H11N9)	**HM849013 (98)** A/mallard/PT/27972-B139/2007 (H9N2)	**CY021510 (99)** A/quail/Italy/4610/2003 (H7N2)	**EU580556 (98)** A/Anas querquedula/Astrakhan/3091/2002 (H4N8)
**H13N2**	**GU982292 (98)** A/great black-headed gull/Atyrau/773/2004 (H13N6)	**AJ574903 (96)** A/Pekin duck/France/92/00 (H6N2)	**GQ907319 (99)** A/Mongolian gull/Mongolia/405/2007 (H13N6)	**CY077004 (98)** A/black-headed gull/Sweden/1/2005 (H13N8)
**H16N3**	**CY043899 (98)** A/shorebird/Delaware/224/2006 (H13N9)	**CY045385 (95)** A/shorebird/Delaware/195/2006 (H16N3)	**AY684908 (98)** A/black-headed gull/Sweden/2/99 (H16N3)	**GQ907298 (98)** A/black headed gull/Mongolia/1756/2006 (H16N3)
